# Machine Learning in Hypertension Detection: A Study on World Hypertension Day Data

**DOI:** 10.1007/s10916-022-01900-5

**Published:** 2022-12-29

**Authors:** Sara Montagna, Martino Francesco Pengo, Stefano Ferretti, Claudio Borghi, Claudio Ferri, Guido Grassi, Maria Lorenza Muiesan, Gianfranco Parati

**Affiliations:** 1 DiSPeA–University of Urbino Carlo Bo, Piazza della Repubblica 13, Urbino, 61029 Italy; 2 Istituto Auxologico Italiano IRCCS, Milan, Italy; 3 SMS–University of Milano Bicocca, Milan, Italy; 4 University of Bologna, Bologna, Italy; 5 MESVA–University of L’Aquila, L’Aquila, Italy; 6 DSCS–University of Brescia, Brescia, Italy; 7 Spedali Civili 1, Brescia, Italy

**Keywords:** Hypertension, Data analysis, Prevention

## Abstract

Many modifiable and non-modifiable risk factors have been associated with hypertension. However, current screening programs are still failing in identifying individuals at higher risk of hypertension. Given the major impact of high blood pressure on cardiovascular events and mortality, there is an urgent need to find new strategies to improve hypertension detection. We aimed to explore whether a machine learning (ML) algorithm can help identifying individuals predictors of hypertension. We analysed the data set generated by the questionnaires administered during the World Hypertension Day from 2015 to 2019. A total of 20206 individuals have been included for analysis. We tested five ML algorithms, exploiting different balancing techniques. Moreover, we computed the performance of the medical protocol currently adopted in the screening programs. Results show that a gain of sensitivity reflects in a loss of specificity, bringing to a scenario where there is not an algorithm and a configuration which properly outperforms against the others. However, Random Forest provides interesting performances (0.818 sensitivity – 0.629 specificity) compared with medical protocols (0.906 sensitivity – 0.230 specificity). Detection of hypertension at a population level still remains challenging and a machine learning approach could help in making screening programs more precise and cost effective, when based on accurate data collection. More studies are needed to identify new features to be acquired and to further improve the performances of ML models.

## Introduction

Arterial hypertension still remains the most important modifiable risk factor for cardiovascular disease worldwide. Despite extensive knowledge about ways to prevent and treat hypertension, the global incidence and prevalence of hypertension and its cardiovascular complications are still elevated mainly due to inadequacies in prevention, detection and control [1, 2]. The high variability characterising blood pressure (BP) values, together with the lack of specific symptoms of this condition, make the detection of hypertension still challenging.

Since 2005, the World Hypertension League has been leading a global campaign to raise awareness of the importance of hypertension through annual screening programs. During the 2018 survey, among 502079 participants found to have hypertension, only 59.5% were aware of having such condition [3]. This evidence confirms that current screening programs conducted by the National Health Services are still failing in detecting appropriately hypertension. Therefore, there is an urgent need to find new strategies to improve hypertension detection at a population level, given that identifying risk factors for hypertension may facilitate earlier interventions, aimed at preventing future development of hypertension, early detecting its appearance and reducing the incidence of its long-term consequences.

In the recent years, artificial intelligence (AI), which includes all computer systems able to perform tasks normally requiring human intelligence, has been successfully applied to healthcare and shown to be a valid tool in managing different clinical conditions [4, 5].

The Italian Society of Hypertension (SIIA) conducts every year a national campaign to increase awareness of the importance of high BP detection. Over the years, several questionnaires related to hypertension have been administered, generating a large dataset. In particular, 37110 individuals participated in the World Hypertension Day campaigns from 2015 to 2019. From the initial dataset, 20206 subjects have been selected for the present study, after removing those with high BP already diagnosed, out of which 4192 (20.75%) with newly discovered hypertension. Data include demographics, risk factor information, questions about general knowledge on hypertension and three measures of systolic and diastolic BP and heart rate. The aim was to apply supervised machine learning (ML) algorithms to such a large dataset in order to find a model capable of detecting unknown hypertension, and comparing their performances with the current screening protocols. Data were split into a training set with 14144 records and a validation set with 6062 samples. We used five ML models – logistic regression, decision tree, random forest, support vector machine and XGBoost – trained performing a 10-fold cross-validation process on the original training set in the first place, and then applying both oversampling and undersampling techniques for managing the imbalanced nature of data.

The performance of the models was evaluated estimating the sensitivity, specificity, accuracy and precision of the different trained models. Results show that, among different ML algorithms and different balancing techniques exploited, there is not an algorithm and a configuration which properly outperforms against the others, even though Random Forest is the most promising one in all the three schemes. The undersampling experiments are those that provide highest sensitivity scores, but a gain in sensitivity often reflects in poor sensitivity and overall accuracy. In particular, the model that best performs in terms of sensitivity was obtained with XGBoost under undersampling scheme, which however showed poor specificity and, consequently, overall low accuracy. The best compromise is given by Random Forest in the undersampling experiment, that provides sensitivity of 0.818, specificity of 0.629, an overall accuracy of 0.681 and AUC of 0.816.

Medical protocols result in a high sensitivity of 0.906 but have a poor specificity of 0.230, thus including in the screening program subjects that likely will not develop hypertension. This result highlights that the current protocols are not optimised and that novel strategies are needed to avoid unnecessary expanses and to reach a larger population.

Future studies should develop and apply new techniques and algorithms with the goal to improve the model performances, possibly evaluating how accurate data acquisition could be enriched with additional new information that may support the automatic prediction of hypertension occurrence.

## Related work

In recent years, AI has been successfully applied to healthcare as a valid medical tool in different clinical conditions [4, 5]. ML in particular is designed for performing high accuracy predictions on individuals’ outcome without explicit programming but based on learning patterns from acquired data.

Different ML algorithms have been applied in the field of hypertension with very heterogeneous results [6–10]. The review by Martinez-Ríos et al. [11] provides a comprehensive analysis of the literature in the field. The conclusion was that ML has proven to be useful in classifying hypertensive subjects, even though there is not a general agreement on which algorithms perform better, which metrics must be measured to evaluate the model and, mostly, which type of data and features must be acquired to replicate the studies and possibly train predictors that outperform the clinical protocols currently adopted.

To be effective, predictions should be based on data that are accurately, easily and massively collected to screen a population whose size is as large as possible. For instance, predictive models based on genetic data make a mass screening not feasible [7]. Promising results are presented in [10] where four ML models were evaluated on 11 easy-to-collect variables, risk factors (such as smoking, drinking, family history) and anthropometric data. However, data were all acquired in the same local hospital, making results possibly not generalisable to individuals living in other areas.

## Materials and methods

### Study subjects

Data were collected from questionnaires administered during the World Hypertension Day from 2015 to 2019. Individuals willing to participate were asked to fill in an anonymous questionnaire and had their BP measured according to the European Society of Hypertension (ESH) standards (3 consecutive BP readings were performed by trained health personnel with validated automated devices after 5 min rest) [3].

Demographic information (age, sex, BMI), self-reported information on cardio- vascular risk factors (hypertension, diabetes, smoking, high cholesterol, kidney disease, family history of cardiovascular diseases), sleep complaints (snoring, witnessed apneas, daytime sleepiness) and prior cardiovascular diseases (previous cardio and cerebrovascular events, previous myocardial infarction) as well as information about the awareness of hypertension and its health consequences were collected through the questionnaire. Questionnaires were administered in medical check-up points in different cities streets and squares, thus involving a very diverse population.

### Preprocessing

**Fig. 1:**
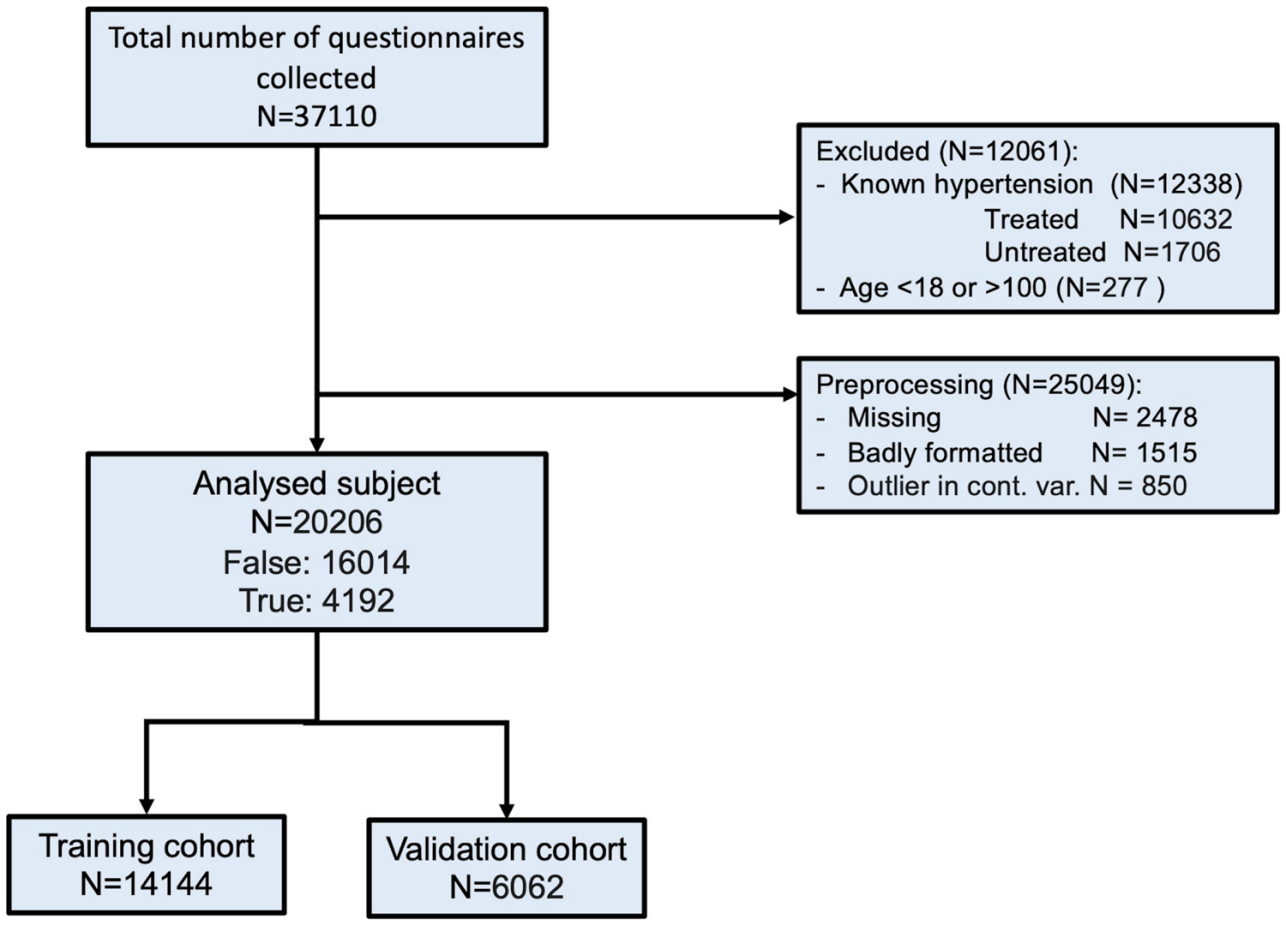
Preprocessing flowchart

In order to analyse the raw data, a set of preprocessing techniques have been applied to the dataset. We included data of adult subjects only (age $\geqslant$ 18 and $\leqslant$ 100 years) with new-onset hypertension. Patients with known hypertension or already treated for hypertension were excluded. Participants were classified as newly detected hypertension if, computed the mean value of the last two BP measurements, at least one between systolic and diastolic BP was equal or greater than 140 or 90 mmHg respectively [12]. Records with missing values have been removed since some of the algorithms adopted in the present study do not support missing values. Outliers for normally distributed data (age, height, weight), defined as those values deviating from the mean more than three times the standard deviation (outside the range $\mu \pm 3 \sigma$) were also excluded from the analysis because were classified as errors in data acquisition. The whole preprocessing procedure is shown in Fig. 1.

Features on top of which our analysis grounds, are listed in Tables 1 and 2. All the features containing categorical values have been converted into a set of dummy variables, each one modelling one of the categories, for a total of 28 dummy variables and 2 continuous ones. For each feature in the table, a statistic description is available, mean/std for continuous variable, or % for dummy variables. Features include the information collected from questionnaires, namely individual’s medical history, subject demographic, clinical characteristics, lifestyle factors, anthropometric measurements, and general knowledge on hypertension.

Accordingly, our dataset is composed of 37110 individuals before applying exclusion criteria, which led to 20206 subjects being included in the analyses. This cohort includes middle aged subjects almost equally distributed in term of sex (51.65% females), with a normal BMI (24.74 kg/m2). Most of the included subjects were in primary prevention as only 3.52% reported a previous cardiovascular event.

**Table 1:** Descriptive statistics for continuous variables (subjects n = 20206)

Feature	Mean	SD	Median
Age	50.89	17.3	52
BMI	24.74	3.86	24.38

**Table 2:** Descriptive statistics for dummy variables. Percentage are of true values

Feature	Percentage
Female	51.65
Cardiovascular risk factors	
smoker	20.11
chronic kidney disease	2.83
diabetes mellitus	4.27
previous heart ischemic events	3.52
high cholesterol	19.23
previous brain ischemic events	1.63
family history of hypertension	20.18
Previous hypertensive emergency	2.37
Sleep complaints	
daytime somnolence	28.12
snoring	29.23
sleep apnea	9.11
Awareness of hypertension health consequences	
heart ischemia	66.54
brain ischemia	54.25
renal insufficiency	76.79
liver insufficiency	87.25
blindness	23.10
diabetes mellitus	15.37
Awareness of habits to prevent hypertension	
low Kcal diet	20.20
low fat and salt diet	65.47
low alcohol	50.06
drink one glass of wine per day	19.23
30 min fitness	62.29
intensive fitness	9.66
no coffee	71.98
no smoking	61.61
periodic medical check	44.76
after symptoms medical check	9.73

### Data analyses with machine learning algorithms

Algorithms have been trained to predict, on top of the features of Tables 1 and 2, an individual’s hypertension risk. Since the goal it to detect hypertension, the database was split into two classes: 16014 subjects (79.25 %) with normal BP and 4192 subjects with newly discovered hypertension (20.75 %).

Due to the imbalance of the two groups, in order to avoid that the trained model overfits on the majority class, a combination of techniques was adopted: *(i)* accuracy was combined with precision and sensitivity to evaluate the model performance and a different coefficient was used to evaluate the errors of the classes *(ii)* the minority class was oversampled, *(iii)* the majority class undersampled. Accordingly, in this study, we performed different experiments in order to find the best predictors and achieve better performance.

Data were randomly split into a training set (70%, n = 14144), used for model construction and development, and a validation set (30%, n = 6062), used to test the performance of the derived model.

As a first experiment, we adopted the imbalanced dataset and we scaled errors with weights inversely proportional to the double of class frequencies in the input data. Given *N* as the samples number, $N_c$ the number of classes in the problem and $N_i$ the number of occurrences of class *i*, the weight $w_i$ to balance class *i* is $w_i = \frac{N}{N_c \times N_i}$

Then, we tested the performances of the algorithms exploiting resampling methods to transform the composition of the training dataset and balance the class distribution. Oversampling consists in creating synthetic samples of the under-represented class, thus generating a training set with two classes equally populated: we adopted to this purpose the SMOTE technique [13] and obtained a training set with 22420 samples (11210 positive samples and 11210 negative samples). Validation has been performed on the same test set as for the previous experiment. Conversely, undersampling consists in reducing the data by eliminating examples belonging to the majority class: we adopted a random strategy to this purpose.

For all the three schemes of experiments, we ran a Grid Search with 10- fold cross-validation to prevent overfitting on the training set and chose the hyperparameters of the prediction model. We chose to adopt sensitivity as the main measure of model to be maximised during the training phase, in order to find the model that optimises the number of positive individuals correctly classified (true positive). The best model built during the training phase has been selected and tested on the validation set. To evaluate the performance of the derived prediction model sensitivity, specificity, precision and accuracy have been computed. Moreover, the receiver operating characteristic (ROC) curve and validated area under the curve (AUC) value were derived.

This workflow has been adopted to evaluate different machine learning algorithms:

1. *Logistic Regression*: a binary statistical model that estimates the probability of an event occurring, based on a given dataset of independent variables. To map predictions and their probabilities, this method uses a sigmoid logistic function of the form $p(x) = \frac{1}{1 + e^{-x}}$ [14];
2. *Decision tree classifier*: a scheme that uses a tree-like model of decisions to classify an input [15];
3. *Random Forest classifier*: an ensemble learning technique that uses a combination of decision trees as the base classifiers. Each tree is constructed from a sample from the original dataset and collected outputs are combined to obtain the final classification [15].
4. *Support vector machine (SVM) classifier*: a class of linear algorithms that generate a hyperplane to separates different classes of data with as wide a margin as possible [14].
5. *XGBoost model*: a scalable, distributed gradient-boosted decision tree that, similarly to random forests, combines multiple machine learning algorithms to obtain a better model [16].

Since the majority of features, available in the dataset, were categorical, we avoided those machine learning algorithms thought to work on numerical values, e.g., K-Nearest Neighbors (K-NN). All analyses were performed using the scikit-learn library [17].

**Table 3:** WHO risk factors

Feature
Age > 65 years
BMI > 25
Smoke
Renal insufficiency
Diabetes mellitus
Previous heart ischemic events
Previous brain ischemic events
High cholesterol
Family history of hypertension
Sleep complaints

### Data analyses with medical protocols

In order to make our evaluation as complete as possible, we formalised the medical protocol extracted from the WHO list of risk factors^1^. The set of rules derived has been applied to the original dataset and a confusion matrix defined, basically reporting how many positive inidviduals were intercepted and how many were left out, as well as how many negative individuals were classified as positive, bringing to overscreening and unnecessary medical tests. In particular, dealing with the features reported in Table 2 if an individual was positive to one of the factors reported Table 3, he/she was classified as positive.

## Results

The clinical goal of this study was to maximise the number of individuals included in the screening who will likely develop hypertension, while minimising included individuals who will not develop hypertension. As such, we needed to select the model that provides the best compromise between sensitivity and specificity.

**Fig. 2:**
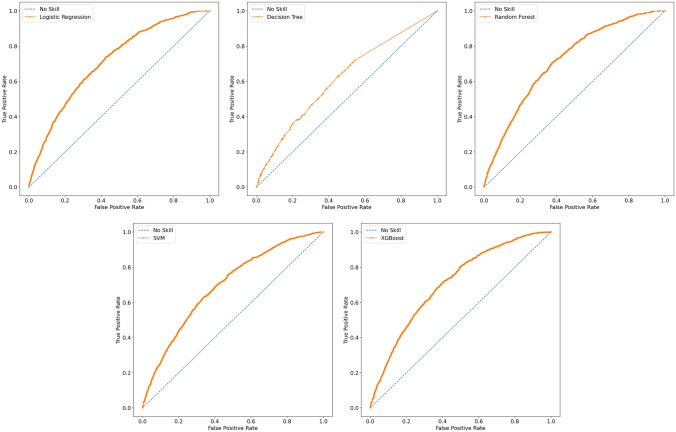
ROC curves for the classification algorithms on the original dataset

The evaluation metrics of the different models, for each of the three schemes evaluated, is presented in Table 4, highlighting the highest measure. Moreover, Fig. 2 shows the ROC curves associated with the best model found for each of the 5 classification algorithms from the original dataset. With the exception of decision tree, which provides the worse performances, from Fig. 2 we derive that, training algorithms on the original dataset, none of the ROC curves resulting from the other models outperforms and AUC remains low, thus requiring further experiments.

**Fig. 3:**
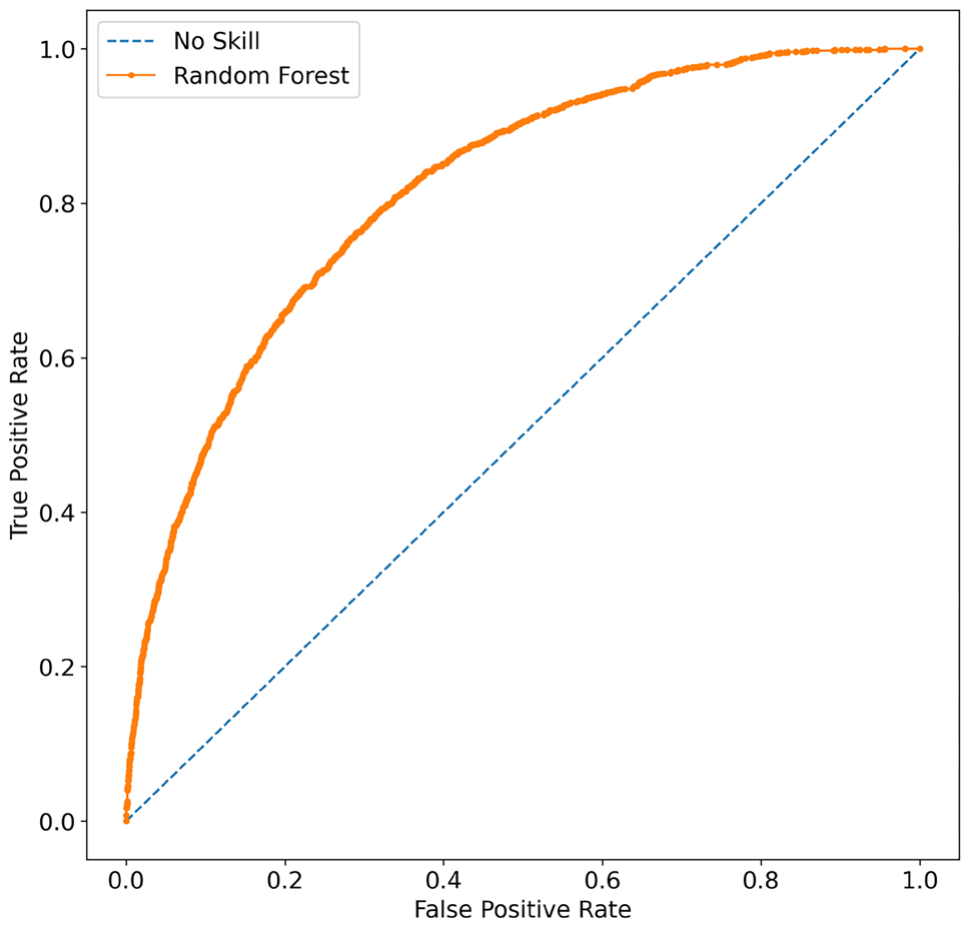
ROC curve for the best performing model, *i.e.,* the Random-Forest in the undersampling experiment

Extending the study with resampling methods, we observe that oversampling significantly drops sensitivity and slightly improves specificity of the different algorithms, while undersampling affects positively both these indicators. Generally speaking, the model with the highest specificity, accuracy and precision was obtained with Random Forest in oversampling scheme. However, the sensitivity was 0.368 leading far more than 50% of positive individuals being classified as negative. XGBoost performs very well with a sensitivity of 0.855 in oversampling, and a very promising 0.988 in undersampling configuration, but specificity falls to 0.404 and 0.113 respectively, resulting in low overall accuracy. The best compromise was achieved with the Random Forest model, getting to a sensitivity of 0.818 and a specificity of 0.629, and an overall accuracy score of 0.681. The corresponding AUC is 0.816, the highest we obtained among the different experiments and models, and the corresponding ROC curve is reported in Fig. 3

When compared with ML algorithms, use of the known risk factors as performed in standard care yielded to a sensitivity of more than 90% but a specificity of 23%, leading to an accuracy of 37%.

**Table 4:** Performance in the test set of the three experimented schemes, original, oversampling and undersampling the training set, and of the medical protocol evaluation

	Original				
Model	Sensitivity	Specificity	Precision	Accuracy	AUC
Logistic Regression	0.666	0.637	0.325	0.644	0.714
Decision Tree	0.524	0.656	0.285	0.629	0.616
Random Forest	0.582	**0.723**	**0.351**	**0.694**	**0.716**
Support Vector Machines	**0.790**	0.483	0.278	0.547	0.619
XGBoost	0.701	0.610	0.320	0.629	0.710

	Oversampling				
Model	Sensitivity	Specificity	Precision	Accuracy	AUC
Logistic Regression	0.497	0.773	0.365	0.716	0.702
Decision Tree	0.352	0.743	0.304	0.698	0.590
Random Forest	0.368	**0.846**	**0.386**	**0.747**	**0.712**
Support Vector Machines	0.629	0.652	0.322	0.647	0.641
XGBoost	**0.855**	0.404	0.273	0.497	0.708

	Undersampling				
Model	Sensitivity	Specificity	Precision	Accuracy	AUC
Logistic Regression	0.678	0.633	0.326	0.642	0.711
Decision Tree	0.739	0.610	0.332	0.637	0.739
Random Forest	0.818	**0.629**	**0.377**	**0.681**	**0.816**
Support Vector Machines	0.754	0.561	0.310	0.601	0.721
XGBoost	**0.988**	0.113	0.226	0.295	0.709

	Medical Protocol				
Model	Sensitivity	Specificity	Precision	Accuracy	AUC
Formal rules	0.906	0.230	0.236	0.370	0.568

In each of the three experiments, and for each indicator, the highest value is marked in order to highlight the best performing model

## Discussion

Screening for hypertension still remains one of the most critical aspects in cardiovascular prevention. Appropriate screening programs could allow early detection of patients at risk and prevent the development of hypertension mediated organ damage. Big data analysis through AI algorithms could improve sensitivity and specificity of such screening programs.

In this study we evaluated and compared five supervised ML algorithms in predicting hypertension based on a large dataset acquired during a national campaign during the World Hypertension Day. Although the best performing model achieved with the Random Forest algorithm allowed identification of hypertensives and normotensives with acceptable accuracy, sensitivity appears to be suboptimal limiting its use in clinical practice. As such, the results of the present work highlight both the advantages and limitations of ML: if on one hand ML automates the entire data analysis, resulting in more comprehensive, deeper, and faster insights, it strongly depends on the quality of data. Despite the huge amount of scientific literature reporting ML applications in healthcare, suggesting that AI applications can improve accuracy and timeliness of diagnoses, results are not always convincing. Have data the sufficient quality to be adopted for ML analyses? Are data containing the information needed for a clinical evaluation? Accordingly, an appropriate and comprehensive data collection is of fundamental importance as predictive models are only as good as the the quality of data from which they are built.

However, adopting the standard approach that includes the evaluation of known risk factors identified by the WHO medical protocol, allowed acceptable sensitivity but a very low specificity, leading to an approach that causes high expanses for the health system and that is not sustainable from an economic point of view.

Nevertheless, integrating the best ML model with clinical algorithms could represent the best compromise that maximises the number of true positives and minimises the false negatives.

## Conclusion

Supervised ML algorithms applied to a big dataset collected in Italy by the SIIA, allowed to identify hypertension with modest accuracy and suboptimal sensitivity. Such an approach, if further improved and tested in different cohorts, could help with hypertension screening and might represent a potentially cost-effective alternative to patients’ evaluation by physicians. Future research should consider the adoption of ML techniques to develop a clinical model which should combine in practice the accuracy in diagnosing hypertension with the need to reduce the costs particularly in low resource settings.

## Data Availability

The participants of this study did not give written consent for their data to be shared publicly, so due to the sensitive nature of the research, supporting data is not available.

## A. Italian investigators

1. Francesco Cipollone - Centro per l’Aterosclerosi, l’Ipertensione Arteriosa AO Chieti
2. Claudio Ferri - Servizio Ambulatoriale per l’Ipertensione Arteriosa e la Prevenzione Cardiovascolare, Ospedale Regionale “San Salvatore” , L’Aquila
3. Francesca Mallamaci - Centro dell’Ipertensone, UO di Nefrologia Dialisi e Trapianto di Rene, Grande Ospedale Metropolitano “Bianchi Melacrino Morelli”, Reggio Calabria
4. Nicola De Luca - Centro Ipertensione, AOU “Federico II”, Napoli
5. Ferruccio Galletti - Medicina Interna, Ipertensione e Prevenzione Cardiovascolare, AOU “Federico II”, Napoli
6. Luciano Di Meo - ASL CE - Centro Ipertensione e Prevenzione Cardiovascolare - Distretto 14, Cellole (CE)
7. Teodoro Marotta - Poliambulatorio “Cesare Battisti” - Distretto 31 - ASL Napoli 1 Centro, Napoli
8. Aniello De Leo - Ambulatorio per l’Ipertensione Arteriosa e la Prevenzione del Rischio Cardiovascolare Ospedale “Fatebenefratelli”, Napoli
9. Giovanni Rosiello - ASL Napoli 1 Centro - UOSD di Patologia Cardiovascolare, Napoli
10. Michele Ciccarelli - AOU “San Giovanni di Dio e Ruggi d’Aragona”, Salerno
11. Claudio Borghi - Centro per la prevenzione e cura dell’Ipertensione Arteriosa, Policlinico “S. Orsola”, Bologna
12. Renzo Roncuzzi - Casa di Cura Villa Erbosa , Bologna
13. Vincenzo Mazzeo - Ambulatorio diagnosi e terapia dell’Ipertensione Arteriosa Presidio Ospedaliero “Pierantoni”, Forlì
14. Claudio Guadagni - Centro per la diagnosi e cura dell’Ipertensione Arteriosa (Polo Sanitario Ravenna 33), Ravenna
15. Leonardo Sechi - Centro Ipertensione, Clinica Medica Università di Udine
16. Bruno Fabris - Centro per lo Studio e la Cura dell’Ipertensione Arteriosa, Trieste
17. Massimo Volpe - Centro per la Diagnosi e Cura dell’Ipertensione Arteriosa, AO “Sant’Andrea”, Roma
18. Claudio Letizia - Centro dell’Ipertensione Secondaria, Centro di Riferimento della Regione Lazio dell’Ipertensione Secondaria ed Endocrinopatie di difficile diagnosi, Università di Roma “Sapienza”, AOU Policlinico “Umberto I” di Roma
19. Dario Manfellotto - Centro Ipertensione Arteriosa e Gestazionale, Ospedale “Fatebenefratelli” Roma
20. Marco Mettimano - Policlinico Universitario “Agostino Gemelli” di Roma
21. Roberto Pontremoli - Centro per la Diagnosi e Cura dell’Ipertensione Arteriosa, AOU “San Martino”, Genova
22. Roberto Ervo - Centro Dialisi Ventimiglia Ospedale Bordighera/Ventimiglia ASL 1 Imperiese, Bordighera
23. Aldo Pende - Centro per l’Ipertensione, AOU “San Martino”, Genova
24. Maria Lorenza Muiesan - Centro per la prevenzione e cura dell’Ipertensione Arteriosa, Università degli Studi di Brescia
25. Cristina Giannattasio - Ambulatorio Ipertensione, Ospedale Niguarda “Ca’ Granda” e Università di Milano-Bicocca, Milano
26. Gianfranco Parati - Istituto Scientifico Ospedale “San Luca” - IRCCS Istituto Auxologico Italiano, Università di Milano-Bicocca, Milano
27. Giuseppe Mancia - Centro Studi Ipertensione e Malattie Vascolari - Policlinico di Monza, Verano Brianza (MB)
28. Rosario Ariano - Ambulatorio per la diagnosi e terapia dell’ipertensione arteriosa, UO Nefrologia AO di Cremona
29. Guido Garavelli - Ambulatorio per Ipertensione Arteriosa, Istituto “Figlie di San Camillo”, Cremona
30. Fabio Albini - Ambulatorio Ipertensione e Protezione Cardiovascolare Milano Nord, Cusano Milanino (MI)
31. Massimo Crippa - Unità semplice di diagnosi e trattamento dell’Ipertensione arteriosa, PO Gardone Val Trompia, AO “Spedali Civili” di Brescia, Gardone Valtrompia (BS)
32. Flavio Acquistapace - GB Mangioni Hospital - GVM Care & Research, Lecco
33. Maria Teresa Lavazza - AO Ospedale di Legnano
34. Antonio Agrati, Giovanni Ferraro - Ambulatorio Medicina Generale Ipertensione Dipartimento Medico Polispecialistico Ospedale “Niguarda Cà Granda” Milano
35. Stefano Carugo - Asst - Santi Paolo e Carlo, Milano
36. Chiara Lonati - Centro Ipertensione Arteriosa, Ospedale Classificato “San Giuseppe”, Milano
37. Maurizio Turiel - Servizio di Cardiologia - IRCCS Istituto Galeazzi, Milano
38. Amedeo Mugellini - Centro per l’Ipertensione e la Fisiopatologia Cardiovascolare - Università degli Studi di Pavia - Sezione di Medicina Interna, Malattie Vascolari e Metaboliche IRCCS Policlinico “San Matteo”, Pavia
39. Claudio Pini - Azienda Socio Sanitaria Territoriale Lariana, San Fermo della Battaglia (CO)
40. Giuseppina Dognini - AO Treviglio, Ospedale Treviglio Caravaggio, SC Medicina Generale, Treviglio
41. Andrea Maresca - Centro per la diagnosi e Terapia dell’Ipertensione Arteriosa, UO Medicina1, Ospedale di Circolo e “Fondazione Macchi” ASST Sette Laghi, Varese
42. Riccardo Sarzani - Centro di Riferimento Regione Marche Ipertensione Arteriosa e Malattie Cardiovascolari Clinica di Medicina Interna e Geriatria Università Politecnica delle Marche e IRCCS-INRCA, Torrette di Ancona, Ancona
43. Giuseppe Lembo - IRCCS Neuromed - Polo Didattico Sede distaccata Molise, “Sapienza” Università di Roma, Pozzilli (IS)
44. Franco Veglio - Centro Ipertensione Arteriosa, Università di Torino, Torino
45. Aldo Ortensia - Ambulatorio per Ipertensione Nefrovascolare, AON “S.S. Antonio e Biagio e Cesare Arrigo” Alessandria
46. Alessandro Rossi - Servizio per Ipertensione Medicina Interna, Ospedale di Chieri, (TO)
47. Pietro Nazzaro - Centro di Prevenzione Cerebrovascolare ed Ipertensione Arteriosa “A.M.Pirrelli”, Bari
48. Giuseppe Ranieri - Centro Ipertensione Arteriosa, AOU Policlinico di Bari
49. Vito Vulpis - UOS Diagnosi e Cura dell’Ipertensione Arteriosa - Medicina Interna Ospedaliera, AUO Policlinico di Bari
50. Nicola Morelli - Presidio Territoriale Assistenziale “F. Jaia”, Conversano (BA)
51. Giuseppe Antonio De Giorgi - Clinica San Francesco s.r.l. - Fondatore Giovanni Tartaro , Galatina (LE)
52. Francesco Cocco - SC Cardiologia Utic - PO Manduria ASL TA, Manduria (TA)
53. Rachele Grifa - Ospedale “Casa Sollievo della Sofferenza” - IRCCS Dipartimento di Scienze Mediche - SC di Nefrologia e Dialisi, San Giovanni Rotondo (FG)
54. Santina Cottone - UO Dipartimentale di Nefrologia ed Ipertensione, Palermo
55. Paola Belluardo - SC Cardiologia - UTIC Ospedale “Maggiore” di Modica, (RG)
56. Stefano Taddei - Centro per la cura e la diagnosi dell’Ipertensione Arteriosa, AOU Pisana, Pisa
57. Salvatore Lenti - Centro Ipertensione Arteriosa di II livello - UOSD Ipertensione, dislipidemia e rischio cardiovascolare - Dipartimento Medicina Interna - Ospedale “San Donato” Arezzo - USL sudest Toscana, Arezzo
58. Andrea Ungar - Centro di Riferimento Regionale per l’Ipertensione Arteriosa dell’anziano della Regione Toscana, Cardiologia e Medicina Geriatrica, AOU “Careggi” e Università di Firenze
59. Franco Cipollini - Ambulatorio Specialistico per l’Ipertensione Arteriosa, UO Medicina Interna, Ospedale “S. Jacopo”, Pistoia, Azienda USL Toscana Centro, Pistoia
60. Stefano Barolo - Medicina Interna Ospedale di Silandro, Silandro (BZ)
61. Giacomo Pucci - Centro Ipertensione Arteriosa - Dipartimento di Medicina, Università degli Studi di Perugia, SC di Medicina Interna, AO “S. Maria”, Terni
62. Maria Sabina Modesti - Ambulatorio per la Diagnosi e la Cura dell’Ipertensione Arteriosa - SC Medicina Interna - Ospedale Regionale “U. Parini” di Aosta
63. Pietro Minuz - UOC Medicina Generale per lo Studio ed il Trattamento della Malattia Ipertensiva, Dipartimento di Medicina Università di Verona ed AOUI Verona Policlinico “GB Rossi”, Verona
64. Francesca Saladini - ULSS 6 Euganea - UO Cardiologia, PO Cittadella-Camposampiero, (PD)
65. Francesco Fallo - Ambulatorio Divisionale e dell’Attività di Ricovero per il Settore dell’Ipertensione Arteriosa, A.O.U. Padova
66. Giancarlo Parisi - Ambulatorio Diagnostica vascolare e prevenzione cardiovascolare UO Medicina, Ospedale “Immacolata Concezione” - ULSS & Euganea, Piove di Sacco (PD)
67. Marcello Rattazzi - Università degli Studi di Padova, Dipartimento di Medicina-DIMED / ULSS 9 di Treviso, Dipartimento di Medicina Interna, SC di Medicina Interna 1, Treviso
68. Cristiana Leprotti - Centro per L’ipertensione Arteriosa, UOSD Venezia
69. Arrigo Cicero - Department of Medical and Surgical Sciences, Univ. Bologna
70. Guido Iaccarino - Department of Advanced Biomedical Sciences, Univ. Naples, Federico II
71. Giuseppe Mulè - Nephrology and Hypertension Unit of Palermo “Paolo Giaccone
72. Carmine Savoia - Department of Clinical and Molecular Medicine, Sapienza Univ., Roma
73. Carmine Arena - Centro Ospedaliero Militare di Milano
74. Susanna Cozzio - Ospedale di Rovereto, (TN)
75. Plinio Fabiani - Ospedale di Viareggio (LU)
76. Anna Fornasiero - Poliambulatori ULSS Berica, Lonigo (VI)
77. Chiara Grasselli - Azienda Ospedaliera S.M. Nuova, Reggio Emilia
78. Paola Mesiano - Ospedale San Giovanni Bosco, Torino
79. Claudio Pascale - Ospedale Cottolengo, Torino
80. Fabio Bracco - Poliambulatorio Cornareto , Carcare (SV)
81. Roberto Marini, Gorizia
82. Paolo Borgheresi - Poliambulatorio territoriale, Arezzo
83. Amalia Mariotti - Ospedale “Di Summa” - Brindisi
84. Antonio La Grotta - UNI-ASTI SS Polo Universitario, Asti
85. Grzegorz Bilo - Istituto Scientifico Ospedale “San Luca” - IRCCS Istituto Auxologico Italiano, Università di Milano-Bicocca, Milano

## Footnotes

1 https://www.who.int/news-room/fact-sheets/detail/hypertension#:~:text=What%20are%20the%20risk%20factors,and%20being%20overweight%20or%20obese
